# Characterization and Genomic Study of Phage vB_EcoS-B2 Infecting Multidrug-Resistant *Escherichia coli*

**DOI:** 10.3389/fmicb.2018.00793

**Published:** 2018-05-04

**Authors:** Yue Xu, Xinyan Yu, Yu Gu, Xu Huang, Genyan Liu, Xiaoqiu Liu

**Affiliations:** ^1^Key Laboratory of Pathogen Biology of Jiangsu Province, Nanjing Medical University, Nanjing, China; ^2^Department of Microbiology, Nanjing Medical University, Nanjing, China; ^3^Department of Laboratory Medicine, The First Affiliated Hospital with Nanjing Medical University, Nanjing, China; ^4^National Key Clinical Department of Laboratory Medicine, Nanjing, China

**Keywords:** bacteriophage vB_EcoS-B2, genome sequence, multi-drug resistance, comparative genome, mass spectrometry

## Abstract

The potential of bacteriophage as an alternative antibacterial agent has been reconsidered for control of pathogenic bacteria due to the widespread occurrence of multi-drug resistance bacteria. More and more lytic phages have been isolated recently. In the present study, we isolated a lytic phage named vB_EcoS-B2 from waste water. VB_EcoS-B2 has an icosahedral symmetry head and a long tail without a contractile sheath, indicating that it belongs to the family *Siphoviridae*. The complete genome of vB_EcoS-B2 is composed of a circular double stranded DNA of 44,283 bp in length, with 54.77% GC content. vB_EcoS-B2 is homologous to 14 relative phages (such as *Escherichia* phage SSL-2009a, *Escherichia* phage JL1, and *Shigella* phage EP23), but most of these phages exhibit different gene arrangement. Our results serve to extend our understanding toward phage evolution of family *Siphoviridae* of coliphages. Sixty-five putative open reading frames were predicted in the complete genome of vB_EcoS-B2. Twenty-one of proteins encoded by vB_EcoS-B2 were determined in phage particles by Mass Spectrometry. Bacteriophage genome and proteome analysis confirmed the lytic nature of vB_EcoS-B2, namely, the absence of toxin-coding genes, islands of pathogenicity, or genes through lysogeny or transduction. Furthermore, vB_EcoS-B2 significantly reduced the growth of *E. coli* MG1655 and also inhibited the growth of several multi-drug resistant clinical stains of *E. coli*. Phage vB_EcoS-B2 can kill some of the MRD *E. coli* entirely, strongly indicating us that it could be one of the components of phage cocktails to treat multi-drug resistant *E. coli*. This phage could be used to interrupt or reduce the spread of multi-drug resistant *E. coli*.

## Introduction

*Escherichia coli* is a gram-negative bacterium, which can cause intestinal (diarrhea) or extraintestinal (urinary tract infection, septicaemia, pneumonia, and meningitis) diseases in humans and animals (Cabal et al., 2016). Current antibiotic treatments used to treat *E. coli often* result in a spread of multidrug resistance (MDR) of *E. coli* (Lepape and Monnet, 2009). For example, *E. coli* strains produce Extended-Spectrum Beta Lactamase and metallo-β-lactamas, enzymes which make *E. coli* become resistant to several antibiotic drugs (Peña et al., 2006; Birgy et al., 2011). Currently, carbapenems appear to be the last available treatment for the severe infections caused by MDR *E. coli*. The recent finding of carbapenemase which hydrolyzes carbapenem may soon lead to cases with no therapeutic issue (Birgy et al., 2011). The emergence of carbapenemase and the lack of efficient antibiotics are alarming, therefore, the well-tolerated, highly effective therapeutic alternatives are urgently needed (Birgy et al., 2011; Castanheira et al., 2017). This has encouraged researchers into returning to use bacteriophages (phages) as a supplement or substitute of antibiotics to treat infection caused by bacterial (Bolocan et al., 2016).

Although phages have been discovered over a century ago, phages continue to have a major impact on modern biological sciences, especially with the growth of interests in the microbiome (Haq et al., 2012; Laanto et al., 2017) and treating multidrug-resistant bacteria (Kutateladze and Adamia, 2010; Bolocan et al., 2016). Many reports described that various pathogenic *E. coli* have been killed by lysis phages (Bourdin et al., 2014). Therapeutic bacteriophages that efficiently lyse the *E. coli* O104:H4 outbreak strain could be selected easily from a phage bank or isolated from the environment (Merabishvili et al., 2012). Phage EC200PP was able to treat sepsis and meningitis infections induced by drug-resistant *E. coli* S242 (Pouillot et al., 2012). Fitzgerald-Hughes's study proved that Extended-Spectrum Beta Lactamase producing *E. coli* strains can be killed by commercially available and laboratory-isolated bacteriophages (Fitzgerald-Hughes et al., 2014). Dufour et al. determined that bacteriophage LM33-P1 can infect β-lactams and fluoroquinolones resistant *E. coli* strains efficiently both *in vitro* and *in vivo* (Dufour et al., 2016). The study on the efficient treatment of *E. coli*-induced pneumonia with two bacteriophages (536 P1 and 536 P7) showed that a combination of antibiotic treatment and phage therapy resulted in a 100% survival rate in VAP-infected mice (Dufour et al., 2015). Therefore, bacteriophages can be used as a supplement for antibiotics to treat the infection caused by bacteria.

Phages are the most abundant biological entities present on earth, providing an unlimited resource for possible phage applications (Srinivasiah et al., 2008). Phages flourish in oceans, soil, wastewater treatment plants, hot-water springs, and animal gut (Srinivasiah et al., 2008; Williamson et al., 2017). *E. coli* phages are commonly isolated from sewage, hospital waste water, polluted rivers, and fecal samples of humans or animals (Jamalludeen et al., 2009; Dalmasso et al., 2016; Snyder et al., 2016; Amarillas et al., 2017). Selection of wide spectrum phages or phages cocktail components for therapeutic preparation are key procedures to overcome the shortage of phage therapy, such as narrow host range of phages and emergence of bacterial resistance to phages (Regeimbal et al., 2016). Constructing the “right” cocktail is essential for achieving the maximum effectiveness of phage therapy (Bourdin et al., 2014; Schooley et al., 2017). It is also essential to have a set of well-characterized phages available for constructing the “right” cocktail to infect the broad range of bacterial pathogens (Sybesma et al., 2016). Therefore, isolating new phages and unraveling their genome sequence seems to be urgently needed to accumulate sufficient phage stocks for phage therapy.

In the present study, we isolated a lytic phage named vB_EcoS-B2 from waste water. Transmission electron microscopy of vB_EcoS-B2 morphology revealed that it belongs to the *Siphoviridae* family. vB_EcoS-B2 significantly reduced the growth of *E. coli* under laboratory conditions. Prior to its application in phage therapy, we determined the genomic sequence and particle proteins of phage vB_EcoS-B2. Our results confirmed that vB_EcoS-B2 does not include virulent genes, islands of pathogenicity, or genes through lysogeny or transduction, which is a good candidate for phage therapy.

## Materials and methods

### Bacterial strains and culture conditions

The bacterial strains used in this study were listed in Table 1. *E. coli* strains BL21 (DE3), DH5α, JM110, TOP10, BW25113, S17-1 were stocks in our lab. Thirty-five clinical isolates of *E. coli* were isolated from clinical samples of patients in the First Affiliated Hospital of Nanjing Medical University, Nanjing, China. All clinical strains which were used for phage host range determination have antibiotic resistance. Twenty-one of them are Extended-Spectrum Beta Lactamase producing stains. Five of them are metallo-β-lactamas (New Delhi metallo-β-lactamase-5) producing stains. All strains were grown in Luria–Bertani (LB) medium at 37°C.

**Table 1:** Host range spectrum of thebacteriophage vB_EcoS-B2.

***E. coli* strain**	**Source**	**Subtype**	**Resistance**	**Lysis or not**
*E. coli* K-12 MG1655				Clear plaque
*E. coli* TOP10				Clear plaque
*E. coli* S17-1				Clear plaque
*E. coli* DH5α				Clear plaque
*E. coli* BW25113				Clear plaque
*E. coli* BL21(DE3)				Clear plaque
*E. coli* JM110				N
25922	ATCC 25922	Non-ESBL		N
35218	ATCC 35218	ESBL		Turbid plaque
389 A6	Urine	ESBL	Aztreonam, cefazolin, ceftazidime, cefatriaxone, sulfamethoxazole, and trimethoprim	Turbid plaque
389 A9	pus and secretion	ESBL	Aztreonam, cefazolin, ceftazidime, sulfamethoxazole, and trimethoprim	N
389 D9	Urine	Non-ESBL	Sulfamethoxazole and trimethoprim	N
389 E6	Urine	Non-ESBL	Levofloxacin	N
389 G4	Urine	Non-ESBL	Amikacin, Ampicillin sulbactam, aztreonam, cefazolin, cefepime, cefotaxime, cefoxitin, ceftazidime, gentamycin, Levofloxacin,	N
389 G6	Urine	Non-ESBL	Levofloxacin	N
389 G7	Urine	ESBL	Amikacin, Ampicillin sulbactam, aztreonam, cefazolin, cefotaxime, Levofloxacin	N
389 J4	Sputum	ESBL	Aztreonam, cefazolin, cefepime, ceftazidime, cefatriaxone,	N
390 A7	Sputum	Non-ESBL	Sulfamethoxazole and trimethoprim	N
390 B6	Urine	Non-ESBL	Sulfamethoxazole and trimethoprim	N
390 G7	Urine	Non-ESBL	Ampicillin sulbactam, cefazolin, gentamycin, Levofloxacin, sulfamethoxazole, and trimethoprim	Clear plaque
390 H2	Urine	Non-ESBL	Ampicillin sulbactam, cefazolin, gentamycin, Levofloxacin, sulfamethoxazole, and trimethoprim	N
390 J2	Urine	Non-ESBL	Levofloxacin, minocycline, sulfamethoxazole, and trimethoprim	N
391 D3	Urine	Non-ESBL	Cefazolin, gentamycin, sulfamethoxazole, and trimethoprim	N
391 G4	Blood	ESBL	Aztreonam, cefazolin, cefepime, ceftazidime, cefatriaxone,	N
393 B7	Urine	ESBL	Amikacin, amoxicillin and clavulanate, Ampicillin sulbactam, aztreonam, cefazolin, cefepime, cefotaxime, cefoxitin, ceftazidime, gentamycin, Levofloxacin, minocycline, sulfamethoxazole, and trimethoprim	N
393 C1	Ascites	ESBL	Ampicillin sulbactam, aztreonam, cefazolin, cefepime, cefotaxime, ceftazidime, gentamycin, sulfamethoxazole and trimethoprim	Clear plaque
393 C8	Urine	ESBL	Cefazolin, cefepime, cefotaxime, Levofloxacin, sulfamethoxazole, and trimethoprim	N
393 D3	Urine	ESBL	Cefazolin, cefepime, cefotaxime, ceftazidime, Levofloxacin,	N
394 F7	Urine	ESBL	Amoxicillin and clavulanate, aztreonam, cefazolin, cefotaxime, cefoxitin, ceftazidime	N
394 G1	Urine	ESBL	Amoxicillin and clavulanate, cefazolin, cefoxitin, imipenem, sulfamethoxazole, and trimethoprim	N
394 H7	Urine	ESBL	Aztreonam, cefepime, cefotaxime, ceftazidime, Levofloxacin	Clear plaque
395 B5	Urine	ESBL	Ampicillin sulbactam, aztreonam, cefazolin, cefepime, cefotaxime, gentamycin, Levofloxacin, sulfamethoxazole, and trimethoprim	N
395 G6	Urine	ESBL	Aztreonam, cefazolin, cefepime, cefotaxime, ceftazidime, gentamycin	N
395 J2	Sputum	ESBL	Ampicillin, gentamycin, Levofloxacin, minocycline	N
396 F3	Sputum	ESBL	Amikacin, ampicillin, gentamycin, Levofloxacin, minocycline	Clear plaque
396 J1	Urine	ESBL	Cefazolin, cefepime, cefotaxime, imipenem, Levofloxacin,	N
396 J5	Urine	ESBL	Aztreonam, cefazolin, cefepime, cefotaxime, imipenem, Levofloxacin	N
397 C8	Sputum	ESBL	Aztreonam, cefazolin, cefepime, ceftazidime, cefatriaxone	N
397 D3	Urine	ESBL	Ampicillin sulbactam, aztreonam, cefazolin, cefepime, cefotaxime, cefoxitin, gentamycin, Levofloxacin, sulfamethoxazole, and trimethoprim	N
37	n.d	NDM-5	Ampicillin/Sulbactam, aztreonam, imipenem, Levofloxacin, piperacillin, Cefuroxime sodium, cefuroxime axetil, sulfamethoxazole, and trimethoprim, ceftazidime, Tazobactam, Meropenem, cefotetan, cefatriaxone, cefazolin, Cefepime plaster	N
40	n.d	NDM-5	Ampicillin/Sulbactam, amikacin, aztreonam, gentamycin, imipenem, Levofloxacin, piperacillin, Cefuroxime sodium, cefuroxime axetil, sulfamethoxazole and trimethoprim, ceftazidime, Tazobactam, tobramycin, Meropenem, cefotetan, cefatriaxone, cefazolin, Cefepime plaster	N
64	n.d	NDM-5	Ampicillin/Sulbactam, gentamycin, imipenem, Levofloxacin, piperacillin, Cefuroxime sodium, cefuroxime axetil, sulfamethoxazole and trimethoprim, ceftazidime, Tazobactam, tobramycin, Meropenem, cefotetan, cefatriaxone, cefazolin, Cefepime plaster	N
69	n.d	NDM-5	Ampicillin/Sulbactam, amikacin, aztreonam, gentamycin, imipenem, Levofloxacin, piperacillin, Cefuroxime sodium, cefuroxime axetil, sulfamethoxazole and trimethoprim, ceftazidime, Tazobactam, tobramycin, Meropenem, cefotetan, cefatriaxone, cefazolin, Cefepime plaster	N
92	n.d	NDM-5	Ampicillin/Sulbactam, gentamycin, imipenem, Levofloxacin, piperacillin, Cefuroxime sodium, cefuroxime axetil, sulfamethoxazole and trimethoprim, ceftazidime, Tazobactam, tobramycin, Meropenem, cefotetan, cefatriaxone, cefazolin, Cefepime plaster	Turbid plaque

Positive results are indicated by “Clear plaque” or “Turbid plaque,” and negative results are indicated by “N”; n.d., no data available; In the column “subtype,” “ESBL” represent “Extended-Spectrum Beta Lactamase,” “NDM-5” represent “New Delhi metallo-β-lactamase-5.”

### Isolation and propagation of bacteriophages

*E. coli* MG1655 was used for isolating and enriching virulent bacteriophage from waste water in Nanjing. Sewage sample was filtered using 0.45 μm pore-size filters (Millipore, USA) to remove bacteria. Filtrates were added to *E. coli* MG1655 culture in early-log-phase at 37°C for 24 h with constant shaking to enrich the phages. Then the culture was centrifuged at 12,000 g for 10 min at 4°C to remove *E. coli* cells. The enriched phage suspension was tested for plaque formation with *E. coli* MG1655 using the double-layer agar plate method. Plaques formed on the plates after 12 h of incubation at 37°C. Single plaque was picked to start the second (and subsequent) round of amplification. The infection cycle was repeated until the plaques were homogeneous. The phages were then amplified and stored at 4°C.

### Purification of phages

Purification of vB_EcoS-B2 was carried out as described previously with slight modifications (Heo et al., 2009). Briefly, *E. coli* MG1655 culture at the early-log-phase (OD600 = 0.4) was infected by phage vB_EcoS-B2 at 37°C for 3 h with shaking. Cell debris was removed by centrifugation (14,000 g, 10 min, 4°C). The supernatant was passed through 0.45-μm-pore-size filters, yielding a crude extract of phage. Then the phage crude extract was concentrated by ultracentrifugation (100,000 g, 2h, 4°C), and the pellet containing phages was suspended in SM buffer (5.8 g/L NaCl, 2 g/L MgSO_4_·7H_2_O, 50 ml/L 1M pH7.5 Tris-HCl). The concentrated suspension was further purified by cesium chloride gradient centrifugation (90,000 g, 20 h, 4°C). The phage zone was collected (about 1 mL) and diluted into 10 ml SM buffer, followed by participation at 200,000 g for 3 h to remove CsCl. Finally, the pellet was resuspended in SM buffer to yield the highly purified vB_EcoS-B2 particles.

### Electron microscopy

The purified phage particles of vB_EcoS-B2 were fixed on a copper grid with a carbon-coated film and negatively stained with 2% (w/v) phosphotungstic acid. The micrographs were taken under FEI Tecnai G2 Spirit Bio TWIN transmission electron microscope at 80 kV.

### Temperature stability

The stability of bacteriophage under different temperature conditions was determined by constant temperature water bath method, a 1.5 mL tube containing 200 μL (approximately 10^10^ pfu/mL) equal volumes of phage were incubated under different temperature (4, 25,37, 45, 50, 55, 60, and 65°C). The phage titer was determined at intervals of 30 min from 0 to180 min, then at 6 and 24 h by the double-layer method. Three independent experiments were done and the value is represented by means.

### Host range analysis

Ability of vB_EcoS-B2 to infect *E. coli* strains was tested. A total of 10^9^ cells were mixed with melted agar, and this mixture was poured on solid agar to make double-layer agar plates. After solidification, 10 μL of phage suspensions (approximately 10^10^ pfu/mL) of bacteriophage stock suspensions was spotted on plates carrying each bacterial strain. After adsorption of the spots, the plates were inverted and incubated for 24 h at 37°C before the degree of lysis was scored (Postic and Finland, 1961). All experiments were conducted according to the standard institutional guidelines of Nanjing Medical University (Nanjing, China). The study was approved by the research and ethics committee of the First Affiliated Hospital of Nanjing Medical University.

### One-step growth curve

One-step growth experiment was carried out as described previously with little modification (Yang et al., 2015). In brief, *E. coli* MG1655 was grown in LB medium until the early-log-phase (1 × 10^8^ CFU/mL). Phage vB_EcoS-B2 was added to *E. coli* MG1655 culture at a multiplicity of infection (MOI) of 10 separately, and allowed to absorb for 10 min at 37°C. Then the mixture was centrifuged at 14, 000 g for 1 min to remove un-adsorbed phages. After washing twice with fresh LB medium, the pellet of infected cells was resuspended in 50 mL of LB medium and the culture was continuously incubated at 37°C. Using double-layer agar plate method, we determined the free bacteriophage count at each time point. The latency period and burst period were obtained directly from these one-step growth curves. The burst size of vB_EcoS-B2 was calculated by dividing the phage titers at plateau phase by the initial number of infective bacterial cells.

### Bacterial challenge assay

For the bacterial challenge assay, 50 mL fresh LB broth was inoculated with an overnight culture of *E. coli* MG1655 (1% inoculum), followed by incubation at 37°C at 220 rpm until the OD600 was about 0.3. vB_EcoS-B2 stock solutions were then added (MOI = 10) to these cultures. Bacterial growth was monitored by measuring the OD600 at various time points. As a negative control, bacterial cultures were inoculated with SM buffer instead of vB_EcoS-B2. OD600 was recorded at 15 min intervals, over a period of 300 min.

### Extraction and sequencing of the vB_EcoS-B2 genome

The purified phage sample was treated with DNase I (New England Biolabs) and RNase A (Tiangen Biotech) for 2 h at 37°C to digest the exogenous DNA and RNA. The preparation was then treated with proteinase K (Tiangen Biotech) for 15 min at 55°C. The phage genome DNA was further prepared with a TIANamp Bacteria DNA Kit (Tiangen Biotech). DNA concentration was determined using a spectrophotometer (Nanodrop Technologies, USA). The vB_EcoS-B2 genomic DNA was sequenced using an Illumina HiSeq 2500 sequencer and reads were assembled into a whole genome using SOAPdenovov2.04 software and GapCloserv1.12.

### Annotation and comparison

Putative open reading frames (ORFs) were predicted using artemis software (http://www.sanger.ac.uk/science/tools/artemis) and Glimmer 3 (Aggarwal and Ramaswamy, 2002), with a threshold of 30 amino acids (aa) as a minimum for the length of protein. Function annotation was performed using the BLAST tools at NCBI (http://blast.ncbi.nlm.nih.gov/Blast.cgi) against the non-redundant protein sequences database. Transfer RNAs (tRNAs) were identified using tRNAscan-SE (v1.23, http://lowelab.ucsc.edu/tRNAscan-SE) and ribosome RNAs (rRNAs) were determined using RNAmmer (v1.2, http://www.cbs.dtu.dk/services/RNAmmer/). DNAman was used to calculate molecular masses and isoelectric points for predicted phage proteins. The whole viral nucleotide sequence similarities between phages were determined by megablast analysis at NCBI. The global alignment of putative amino acid sequences was carried out by EMBOSS Needle tool at EMBL-EBI (European Molecular Biology Laboratory-European Bioinformatics Institute). Comparison of ORFs from relative phages was performed using EasyFig (http://mjsull.github.io/Easyfig/files.html) (Sullivan et al., 2011). Phylogenetic analyses between the genomes of related phages were performed with MEGA using the Neighbor-Joining algorithm.

### Structural protein analysis of vB_EcoS-B2

The highly purified phage sample was subjected to sodium dodecyl sulfate polyacrylamide gel electrophoresis (SDS-PAGE) using 12% acrylamide concentration. Gels were stained with silver as described by Shevchenko et al. (1996). For protein identification by liquid chromatography electrospray ionization with tandem mass spectrometry (LC-ESI MS/MS), the phage particles were digested with trypsin, and the tryptic peptides were analyzed by Q Exactive mass spectrometer (Thermo Scientific, USA). The corresponding ORFs were searched using MASCOT engine (Matrix Science, London, UK; version 2.2) against the protein sequence library of vB_EcoS-B2.

### Nucleotide sequence accession number

The genome sequence of vB_EcoS-B2 was deposited in GenBank under the accession number MG581355.

## Results and discussion

### Phage morphology

**Figure 1:**
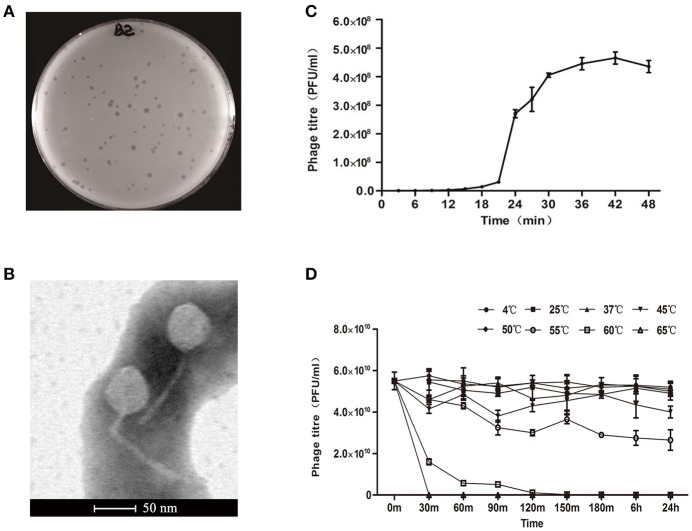
Isolation, morphology, and biological properties of phage vB_EcoS-B2. **(A)** Plaque morphology of phage vB_EcoS-B2. **(B)** Transmission electron micrographs of *E. coli* phage vB_EcoS-B2. Bar indicates 50 nm. **(C)** One-step growth curve of vB_EcoS-B2 on *E. coli* strain MG1655 at 37°C. **(D)** Thermo stability of vB_EcoS-B2: the phages were incubated at different temperatures for 24 h. Each value is the average from three different cultures ± standard deviation in **(C,D)**.

A new *E. coli* phage vB_EcoS-B2 was isolated from wastewater in Nanjing. The phage formed clear round plaque (about 1–3 mm-diameter) with *E. coli* strain MG1655 after overnight culture at 37°C (Figure 1A). The purified phage particles of vB_EcoS-B2 were examined under the Transmission Electron Microscope. vB_EcoS-B2 has an icosahedral symmetry head and a long tail without a contractile sheath, indicating that it belongs to the family of *Siphoviridae*. The isometric head of vB_EcoS-B2 had a mean diameter of 48 nm, and the long non-contractile tail was about 143 ± 6 nm (Figure 1B). vB_EcoS-B2 is closely resembled to the morphology of *Enterobacteria* phage SSL-2009a (an icosahedral head 62 nm in diameter and a long, flexible tail 138 nm in length) and *Shigella* phage EP23 (head diameter 59 ± 3 nm; non-contractile, filamentous tails 142 ± 32 nm in length) (Li et al., 2010; Chang and Kim, 2011).

### Phage population dynamics

One-step growth experiments were performed to assess the population kinetics of vB_EcoS-B2, in the presence of *E. coli* strain MG1655 (Figure 1C). vB_EcoS-B2 had latent period about 20 min. All phages had been released by 30 min after infection (Figure 1C), and had a burst size of 224.1 ± 10.7 phage particles. Latent time of vB_EcoS-B2 was shorter than that of phage EP23 which had a latent period of 45 min (Chang and Kim, 2011). Latent and burst periods of vB_EcoS-B2 were very similar to that of phage SSL-2009a which have latent and burst periods 10–15 and 30–40 min (Li et al., 2010), respectively. Even though the burst size of vB_EcoS-B2 is less than that of SSL-2009a (about 375 PFU per infected cell) (Li et al., 2010), it is still a large burst size. As an antibacterial agent, a phage with a large burst size may indicate an easy selective advantage since phages with large burst sizes can increase the initial dose of phages several hundred-folds in short periods of time (Nilsson, 2014). The phages to be used in phage therapy must be strictly virulent and should be reproduced effectively and rapidly (Nilsson, 2014). Therefore, the large burst size and short latent time of vB_EcoS-B2 make it a good candidate for being used as the biocontrol agent against bacterial pathogens.

### Thermal stability

The thermal stability test was determined at different temperatures (4, 25, 37, 45, 50, 55, 60, and 65°C) whithin 24 h (Figure 1D). Results showed that the biological activity of phage vB_EcoS-B2 did not show any difference within the temperature ranging from 4 to 50°C, but decreased sharply when the temperature increasing above 55°C. Even though vB_EcoS-B2 does not like SSL-2009a which keeps activity more than 45 min over 63°C (Li et al., 2010), vB_EcoS-B2 is stable at relatively low temperature for longer time (Supplementary Figures 1, 2). The phage is stable over a range of temperatures (4–50°C) for 24 h, suggesting that it has a good thermal stability and therefore easy to be preserved.

### Host range and effect of phages on *E. coli* growing culture

The ability of newly isolated phage to lyse *E. coli* strains was assayed by the spot test. vB_EcoS-B2 can infect some of the well-known *E. coli* strains and several clinical MRD *E. coli* stains (Table 1). vB_EcoS-B2 is similar to SSL-2009a that is able to infect some engineered *E. coli* strains (Table 1). vB_EcoS-B2 can also infect the *E. coli* strain ATCC 35218, which could not be infected by SSL-2009a (Li et al., 2010). JL1 is a phage which can infect *E. coli* O157:H7 (Pan et al., 2013). EP23 can infect the three *E. coli* strains and two *S. sonnei* strains (Chang and Kim, 2011). The observations suggested that these very similar phages have different host range. Phage vB_EcoS-B2 only targeted seven MDR *E. coli* strains out of the 35 clinical isolates (Table 1). Therefore, Phage vB_EcoS-B2 has a relative narrow host spectrum. To overcome the narrow host range of phages, it is generally accepted that cocktails of multiple phages can be used to kill the great diversity of *E. coli* strains (Schmerer et al., 2014).

**Figure 2:**
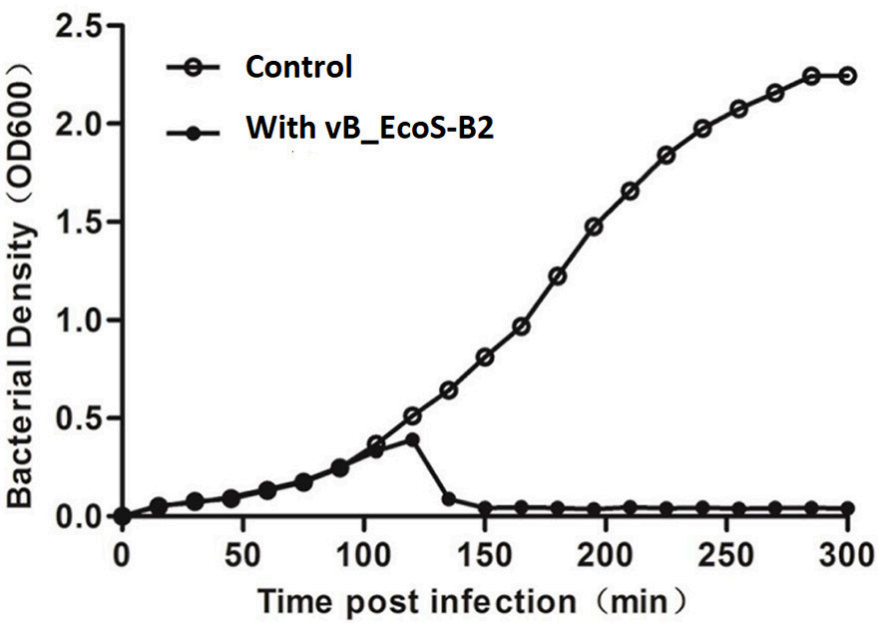
Bacterial challenge assay with phage vB_EcoS-B2 to *E. coli* MG1655. vB_EcoS-B2 was added at an MOI of 10 to the bacterial culture after 1.5 h incubation (OD600 = 0.25) (close circles). OD600 was recorded at 15 min intervals, over a period of 300 min. A bacterial culture inoculated with SM buffer instead of vB_EcoS-B2 was used as the negative control (open circles). Each value is the average from three different cultures.

The effect of vB_EcoS-B2 alone on the growth of strain MG1655 was tested at MOI = 10 after 300 min of the phage added into the strain. vB_EcoS-B2 can efficiently reduce the growth of *E. coli* MG1655 after 30 min of infection (Figure 2, Supplementary Figure 3). Even though its host-range is relatively narrow, vB_EcoS-B2 has the possibility to be used as biocontrol agents against *E. coli* since it had strong lytic ability to *E. coli*. Therefore, it is essential to analyze complete sequence of vB_EcoS-B2 to ensure that the genome of vB_EcoS-B2 did not encode any genes associated with toxins, virulence factors, or lysogenic proteins.

### Basic characteristics of vB_EcoS-B2 genome

**Figure 3:**
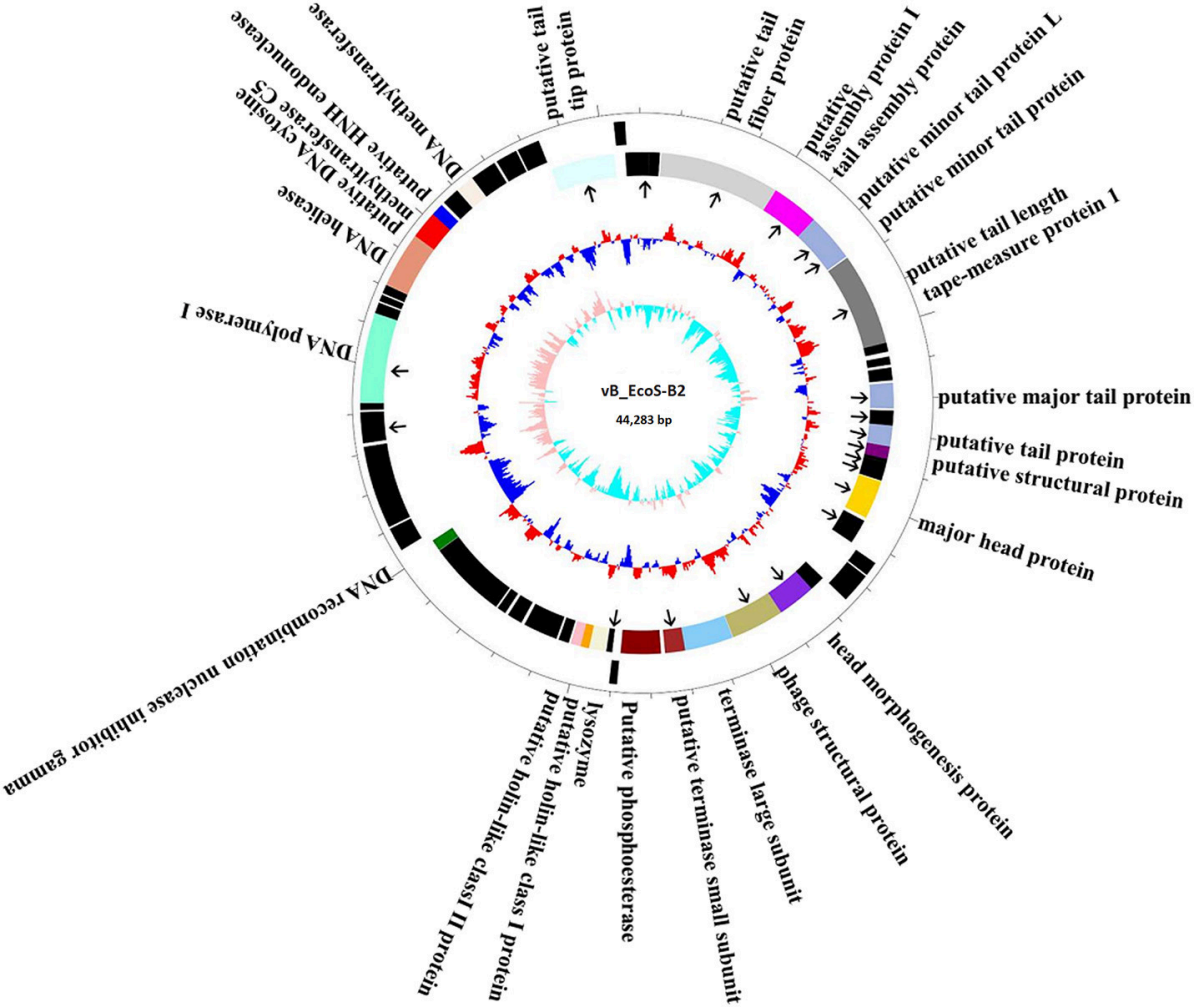
Map of the genome organization of bacteriophage vB_EcoS-B2. The predicted ORFs are indicated as different colors in first circle. The outward showed the forward transcription genes and the inward showed the reversed transcription genes. Different colors identify ORFs with predicted molecular function. Hypothetical proteins marked by black. The second circle shows the G/C content. Red outward indicated that the G/C content of this region is higher than the average G/C content of the whole genome, and blue inward indicated G/C content of this region less than the average. The third circle shows the GC skew. Particle proteins identified by Mass spectrometry were pointed by black arrows.

The complete genome of vB_EcoS-B2 is composed of a circular double stranded DNA of 44,283 bp in length, with 54.77% GC content which was slightly higher than those of *E. coli* (50.6%). Sixty-five putative open reading frames (ORFs) were predicted in the complete genome of vB_EcoS-B2. The annotation of the properties of phage vB_EcoS-B2 genome, such as positions, directions, and putative functions of each gene were summarized in Table 2. Twenty-three ORFs were in the direct strand of the phage genome and 42 of them were in complementary strand (Figure 3). Only 25 ORFs (38.5%) were predicted and determined to be putative functional (different colors), whereas 40 ORFs were assigned to hypothetical proteins (black color) (Figure 3). The 25 predicted functional proteins were categorized into five functional groups: DNA replication/modification (DNA polymerase I, DNA helicase, putative DNA cytosine methyltransferase C5, putative HNH endonuclease, DNA methyltransferase, DNA recombination nuclease inhibitor gamma), host lysis (lysozyme, putative holin-like class I protein, and putative holin-like class II protein), packaging (terminase large subunit, putative terminase small subunit), structural proteins (putative tail tip protein, putative tail fiber protein, putative tail assembly protein I, tail assembly protein, putative minor tail protein L, putative minor tail protein, putative tail length tape-measure protein 1, putative major tail protein, putative tail protein, putative structural protein, major head protein, head morphogenesis protein, and phage structural protein), and additional functions (putative phosphoesterase) (Table 2, Figure 3). In addition, in vB_EcoS-B2 genome no statistically significant BLASTP similarity was identified in genes encoding integrase, recombinase, repressor, and excisionase (markers of temperate bacteriophages) (Carrias et al., 2011). Consequently, the vB_EcoS-B2 phage should be considered as a virulent bacteriophage. Genome analysis also suggests that the phage vB_EcoS-B2 does not encode genes associated with toxins or other virulence factors. Virulent characteristics and no possible pathogen factors make it feasible to be a potential candidate for therapeutic application.

**Table 2:** Predicted ORFs and genes encoded by the vB_EcoS-B2 genome.

**ORFs**	**Start**	**Stop**	**Directions**	**No. of residues**	**MW(da)**	**pI**	**Predicted molecular function**
ORF1	1	2,292	+	763	86167.7	8.56	DNA polymerase I
ORF2	2,292	2,570	+	92	10147.2	10.39	Hypothetical protein
ORF3	2,611	2,784	+	57	6825.7	10.35	Hypothetical protein
ORF4	2,822	3,046	+	74	8761.5	9.94	Hypothetical protein
ORF5	3,051	4,475	+	474	53461.8	9.55	DNA helicase
ORF6	4,472	5,167	+	231	26496.7	8.27	Putative DNA cytosine methyltransferase C5
ORF7	5,164	5,487	+	107	11695	9.14	Putative HNH endonuclease
ORF8	5,569	6,033	+	154	17882.3	8.19	Hypothetical protein
ORF9	6,020	6,505	+	161	18708.4	9.72	DNA methyltransferase
ORF10	6,486	6,710	+	74	8686.6	8.9	Hypothetical protein
ORF11	6,707	7,177	+	156	17872.8	6.25	Hypothetical protein
ORF12	7,242	7,796	+	184	20488.5	4.93	Hypothetical protein
ORF13	7,839	8,414	+	191	21491.9	6.78	Hypothetical protein
ORF14	8,442	10,265	–	607	65194.4	4.79	Putative tail tip protein
ORF15	10,346	10,630	+	94	10251.1	7.28	Hypothetical protein
ORF16	10,582	11,253	–	223	23211.6	8.18	Hypothetical protein
ORF17	11,256	11558	–	100	10216.6	8.5	Hypothetical protein
ORF18	11,596	15,015	–	1,139	125892.9	5.11	Putative tail fiber protein
ORF19	15,012	15629	–	205	21245.4	8.71	Putative tail assembly protein I
ORF20	15,620	16,360	–	246	27577.5	4.95	Tail assembly protein
ORF21	16,363	17,151	–	262	28806.3	4.88	Putative minor tail protein L
ORF22	17,148	17,747	–	199	21696.3	6.52	Putative minor tail protein
ORF23	17,784	20,426	–	880	93307.2	9.93	Putative tail length tape-measure protein 1
ORF24	20,426	20,518	–	30	3341.4	10.5	Hypothetical protein
ORF25	20,490	20,681	–	63	6931.5	8.51	Hypothetical protein
ORF26	20,821	21,051	–	76	8543.3	5.77	Hypothetical protein
ORF27	21,135	21,497	–	120	13958.5	4.78	Hypothetical protein
ORF28	21,567	22,292	–	241	25755.3	5.82	Putative major tail protein
ORF29	22,354	22,776	–	140	15087.1	4.73	Hypothetical protein
ORF30	22,776	23,369	–	197	21918	11.19	Putative tail protein
ORF31	23,371	23,730	–	119	13053.8	9.59	Putative structural protein
ORF32	23,711	23,827	–	38	3990.7	3.76	Hypothetical protein
ORF33	23,829	24,359	–	176	18924.7	3.89	Hypothetical protein
ORF34	24,388	25,488	–	366	38395.4	7.66	Major head protein
ORF35	25,586	26,287	–	233	25384.1	5.51	Hypothetical protein
ORF36	26,388	26,756	+	122	13128.6	5.65	Hypothetical protein
ORF37	26,796	27,560	+	254	28937.1	8.29	Hypothetical protein
ORF38	27,574	27,732	–	52	5739.7	4.33	Hypothetical protein
ORF39	27,686	28,015	–	109	11931.4	9.88	Hypothetical protein
ORF40	28,008	29,111	–	367	40988.6	9.94	Head morphogenesis protein
ORF41	29,095	30,615	–	506	55192.3	4.51	Phage structural protein
ORF42	30,627	32,012	–	461	52196.7	7.52	Terminase large subunit
ORF43	32,012	32,584	–	190	21084	8.05	Putative terminase small subunit
ORF44	32,694	33,836	–	380	42737.4	7.35	Putative phosphoesterase
ORF45	33,857	34,057	+	66	7221.1	8.22	Hypothetical protein
ORF46	34,074	34,241	–	55	5603.2	4.48	Hypothetical protein
ORF47	34,258	34,749	–	163	18156.3	9.41	Lysozyme
ORF48	34,736	34,981	–	81	8870	8.51	Putative holin-like class I protein
ORF49	34,978	35,268	–	96	10183.1	8.23	Putative holin-like class II protein
ORF50	35,323	35,622	–	99	11796	9.72	Hypothetical protein
ORF51	35,687	35,833	–	48	5626.5	11.71	Hypothetical protein
ORF52	35,830	36,363	–	177	20721.2	10.59	Hypothetical protein
ORF53	36,360	36,494	–	44	5107.7	3.43	Hypothetical protein
ORF54	36,485	36,670	–	61	7413.4	11.48	Hypothetical protein
ORF55	36,770	37,075	–	101	11091.8	10.31	Hypothetical protein
ORF56	37,072	37,206	–	44	5206.2	8.24	Hypothetical protein
ORF57	37,297	37,554	–	85	9233.9	11.49	Hypothetical protein
ORF58	37,609	39,861	–	750	82443.1	6.15	Hypothetical protein
ORF59	39,872	40,201	–	109	12545.1	8.47	DNA recombination nuclease inhibitor gamma
ORF60	40,424	41,032	+	202	23053.6	4.52	Hypothetical protein
ORF61	41,081	41,332	+	83	9953.6	9.91	Hypothetical protein
ORF62	41,333	41,521	+	62	7168	8.22	Hypothetical protein
ORF63	41,521	42,951	+	476	52319.8	8.73	Hypothetical protein
ORF64	42,944	43,186	+	80	9323.4	6.49	Hypothetical protein
ORF65	43,277	44,059	+	260	29909.5	4.55	Hypothetical protein

### Structural proteins of vB_EcoS-B2

**Figure 4:**
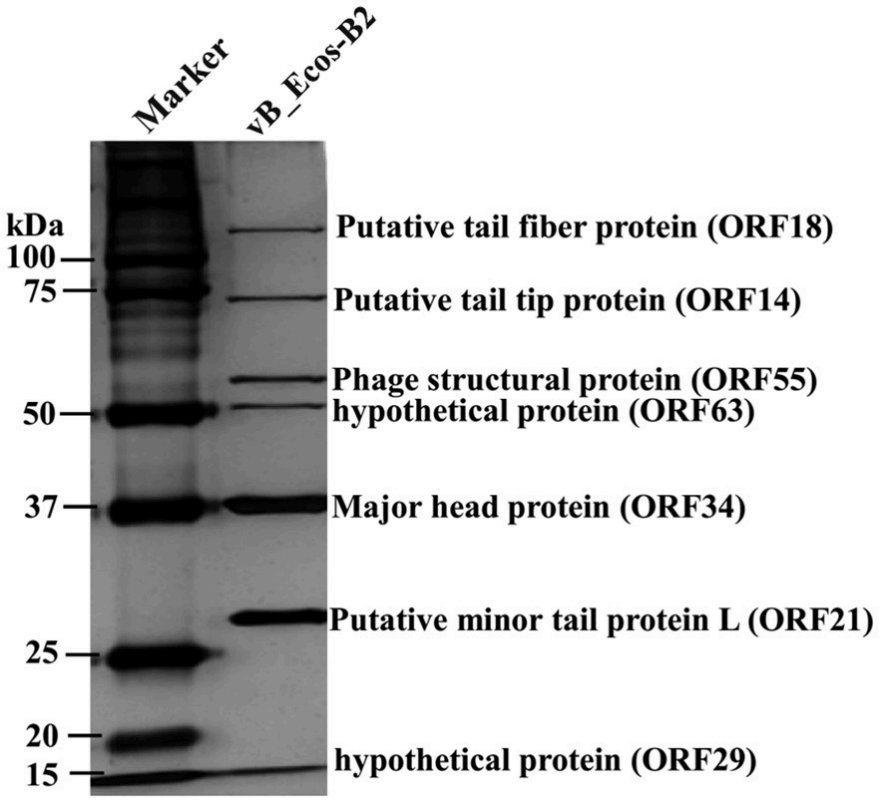
vB_EcoS-B2 virion structural proteins. The purified vB_EcoS-B2 particles were denatured and separated by SDS-PAGE and stained with silver. The positions of seven bands corresponding to different structure proteins are indicated on the right. Molecular mass markers are shown on the left.

In order to analyze the structural proteins of vB_EcoS-B2, purified phage particles were denatured by boiling with sample buffer, and then separated by SDS-PAGE. At least seven distinct protein bands, with molecular weights ranging from 28.8 to 125.9 kDa, were visualized in the SDS-PAGE gel (Figure 4). Five bands were identified as phage structural proteins (Major head protein, Putative minor tail protein L, Phage structural protein, Putative tail tip protein, and Putative tail fiber protein), and two bands were identified as hypothetical proteins (Figure 4). To determine every structural protein, phage particle proteins were identified by mass spectrometry, and results were listed in Table 3. Twenty-one proteins were identified in mass spectrometry, including proteins corresponding to all seven distinct bands on SDS-PAGE gel (Table 3, Figure 4). Twelve out of 13 known structural proteins were identified by mass spectrometry, and some hypothetical proteins (ORF16, ORF29, ORF32, ORF33, ORF35, ORF46, ORF63) were also identified, which may be some unknown structural proteins. Interestingly, DNA polymerase I and putative phosphoesterase were determined in our phage particles. DNA polymerase I could fill DNA gaps during DNA repair, recombination, and replication (Andraos et al., 2004). DNA polymerase I and putative phosphoesterase may play important roles in the phage early infection processes, which will be our interests in our further studies.

**Table 3:** Mass spectrometry data for vB_EcoS-B2.

**ORFs**	**Predicted molecular function**	**Peptides**	**Unique peptides**	**Unique sequence coverage [%]**	**MW(da)**
ORF23	Putative tail length tape-measure protein 1	70	70	77.4	93307.2
ORF18	Putative tail fiber protein	52	52	59.2	125892.9
ORF34	Major head protein	40	40	86.1	38395.4
ORF14	Putative tail tip protein	36	36	56.7	65194.4
ORF41	Phage structural protein	31	31	72.7	55192.3
ORF28	Putative major tail protein	25	25	83.9	25755.3
ORF40	Head morphogenesis protein	24	24	61	40988.6
ORF21	Putative minor tail protein L	18	18	63.4	28806.3
ORF44	Putative phosphoesterase	13	13	36.6	42737.4
ORF35	Hypothetical protein	13	13	56.2	25384.1
ORF22	Putative minor tail protein	11	11	51.5	21696.3
ORF30	Putative tail protein	9	9	43.1	21918
ORF33	Hypothetical protein	7	7	54.2	18924.7
ORF31	Putative structural protein	7	7	58.7	13053.8
ORF46	Hypothetical protein	5	5	94.5	5603.2
ORF16	Hypothetical protein	5	5	38.6	23211.6
ORF63	Hypothetical protein	4	4	15.4	52319.8
ORF29	Hypothetical protein	3	3	41.1	15087.1
ORF1	DNA polymerase I	3	3	8.5	86167.7
ORF32	Hypothetical protein	2	2	22.3	3990.7
ORF19	Putative tail assembly protein I	2	2	21	21245.4

*Particles proteins detected by MS are listed with their predicted functions and molecular mass. Molecular mass was calculated from the amino acid sequence of each gene. Number of identified peptides and unique peptides in each protein and the corresponding unique peptide sequence coverage are also indicated*.

### Comparative genome analysis

**Figure 5:**
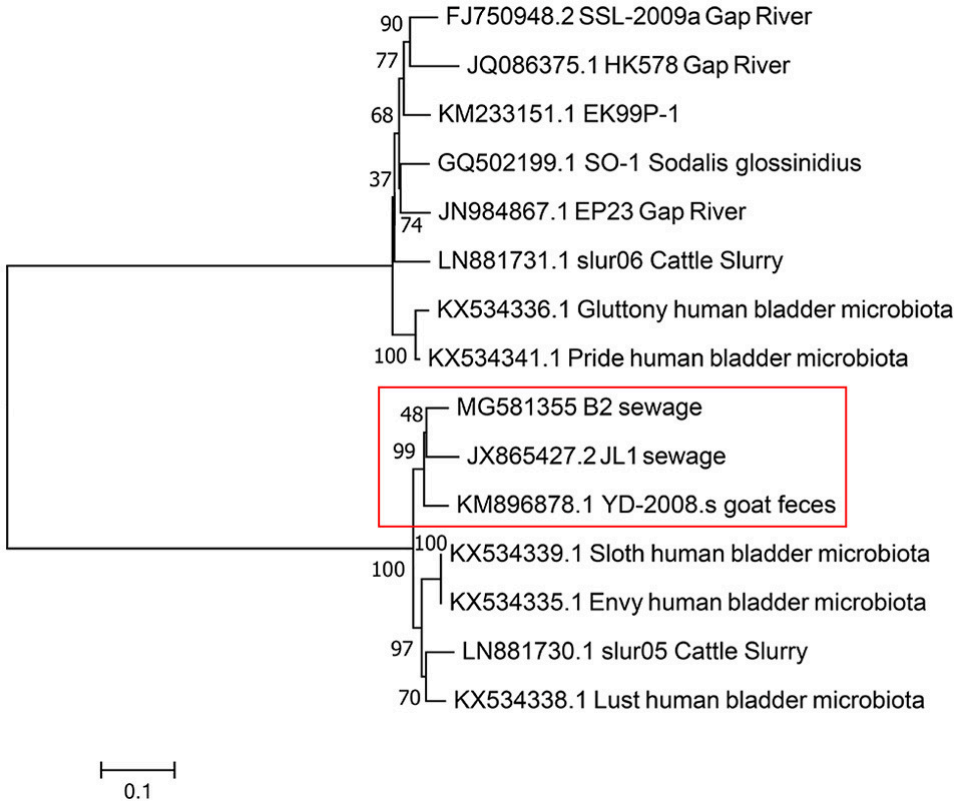
Phylogenetic tree based on whole genome sequence comparisons of selected phages. Whole genome sequence comparison was compared using the ClustalW program, and the phylogenetic tree was generated using the neighbor-joining method with 1,000 bootstrap replicates. vB_EcoS-B2 in brief is“B2”in this phylogenetic trees.

Based on the result of BLAST analyses, the genome sequence of vB_EcoS-B2 displays significant similarity (coverage 87–95%, identity 90–94%) to 14 phages isolated from different regions around the world, suggesting that the complex evolutionary relationships exist among these phages. The corresponding genome sequences of these phages were aligned, concatenated, and a phylogenetic tree was built using the maximum likelihood method (Figure 5). Phylogenetic tree of these phages has two main branches. Phylogenetic tree analysis showed that vB_EcoS-B2 is a novel bacteriophage that is closely related to phages JL1, YD-2008.s, Sloth, Envy, slur05, and lust, and relatively distant to phages SSL-2009a, HK578, EK99P-1, Sodalis phage SO-1, Shigella phage EP23, slur06, Gluttony, and Pride (Figure 5). The results of the comparative genomic analyses extend our understanding of the evolution and relationship between vB_EcoS-B2 and its bacteriophage relatives.

**Figure 6:**
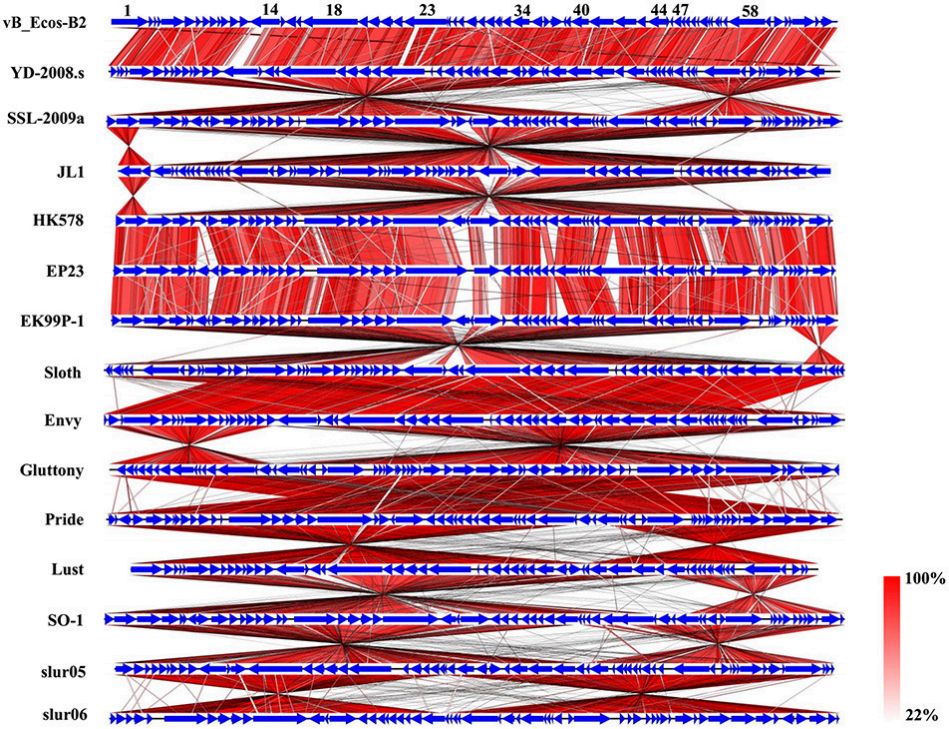
Schematic genomic alignment of the phage vB_EcoS-B2 with other 14 homologous phages. Homologous ORFs or genes are present in blue, and the percentages of amino acid identities are shown with different colors.

Most of the proteins from vB_EcoS-B2 and 14 relative phages are homology with each other. But some blank regions could be observed though genome comparison of these phages (Figure 6). The phage tail tip protein encoded by ORF14 shows a greater divergence between these phages (43–99% coverage and 68–85% identity) (Figure 6). The ORF14 is relatively similar to tail fiber protein of phage Envy (99% coverage and 70% identity). The tail proteins are thought to be involved in host recognition, and confer the phage host range specificity (Hashemolhosseini et al., 1994). The small differences in tail fiber proteins are often associated with significant differences in host ranges and other biological properties (Yosef et al., 2017). This putative tail tip protein of vB_EcoS-B2 is relatively different from tail tip/fiber proteins of other phages, which may make the host range of phage vB_EcoS-B2 different from other relative phages.

Multiple alignment of the vB_EcoS-B2 and 14 relative phages showed that most of regions are highly homologous at protein levels, but they exhibit different gene arrangement with each other (Figure 6). For example, DNA polymerase I and DNA helicase regions of vB_EcoS-B2 were reversely matched with phage SSL-2009a. The tail protein region of phage vB_EcoS-B2 was forwardly matched with the same regions of Enterobacteria phage SSL-2009a. But head morphogenesis protein, terminase large subunit, and lysozyme regions, were back to reversely matched with phage SSL-2009a (Figure 6). Pan et al. found that the gene arrangement and genome structure of phage JL1 are different from those of the other four phages, such as SSL-2009a (Pan et al., 2013). EP23 and SSL-2009a had high similarities in amino acid sequences (95.5% on average). But their gene orders were not conserved with each other (Chang and Kim, 2011). A lot of gene inversions and rearrangement were observed between these phages, such as YD-2008s and SSL-2009a, JL1 and HK578, EK99P-1 and sloth, Envy and Gluttony, Pride and lust, SO-1and slur05 (Figure 6). Gene recombination seems to have been occurred in high frequency in these phages (Figure 6). Our results convinced that topological rearrangement of genomes has lower barriers than changes of amino acid sequences during evolution of phage vB_EcoS-B2 with its phage relatives (Li et al., 2010; Chang and Kim, 2011; Pan et al., 2013). Gene order has major effects on growth rate for T7 phage even when genome entry is normal, suggesting that the consequence of altered gene order probably extends to fitness measures that are not tied to a short generation time (Cecchini et al., 2013). vB_EcoS-B2 and its relatives may be co-evoluted with their host through rearrangement of their genes under different selection pressures. Tailed bacteriophages constitute the most abundant and diverse group of dsDNA viruses, in which evolution is very complicated (Iranzo et al., 2016). Several results illustrated that phages may undergo genetic exchange by horizontal gene transfer from a large shared pool, and that horizontal gene transfer between phages is a component of evolution (Håggard-Ljungquist et al., 1992; Hendrix et al., 1999; Casjens, 2008; Dekel-Bird et al., 2013; Chen et al., 2016). In our study, both horizontal exchange and vertical gene order rearrangement may affect the organization of bacteriophage genomes and blur phylogenetic reconstructions in vB_EcoS-B2 and its relative phages.

In conclusion, we have isolated and characterized a new lytic phage vB_EcoS-B2 which belongs to family *Siphoviridae*, with lytic activity against several MDR *E. coli* isolates. vB_EcoS-B2, like JL1 and EP23, can be assigned into virulent phages because of the presence of lysis genes such as lysozyme, putative holin-like class I protein and putative holin-like class II protein, and no similarities to lysogenic genes coding integrase, repressor, and anti-repressor proteins. The genome sequence analysis of vB_EcoS-B2 provided no evidence of genes related to potential virulence factors or antibiotic resistance genes. In addition, the identification of the 21 structural proteins confirmed that the vB_EcoS-B2 is a new virulent bacteriophage of *E. coli*. It also demonstrated a high degree of identity of vB_EcoS-B2 with ORFs from some other phages. Genome and proteome analysis confirmed the lytic nature of the vB_EcoS-B2. Comparative genome analysis sheds light on the mechanisms of evolutionary changes of these phage genomes. Our results indicate that gene arrangement and genome structure of phage vB_EcoS-B2 is different from that of its phage relatives. The vB_EcoS-B2 genome encodes several putative proteins, including enzymes with antimicrobial activity for the biocontrol of pathogenic bacteria or involved in the phage infection process. The phage genome sequence data also provide useful basic information for further research on the interaction between phages and their hosts.

## Author contributions

YX, XY, YG, and XL conceived, designed and coordinated the study. YX, XY, and YG carried out the experimentation. YX, XY, YG, and XL analyzed the results. GL, XH, YX, XY, YG, and XL contributed reagents, materials, analysis tools. All authors wrote, read, and approved the final manuscript.

### Conflict of interest statement

The authors declare that the research was conducted in the absence of any commercial or financial relationships that could be construed as a potential conflict of interest.
