# Supplementary material for: Characterization and Genomic Study of Phage vB_EcoS-B2 Infecting Multidrug-Resistant Escherichia coli
Source: Front Microbiol. 2018 May 4;9:793. doi: 10.3389/fmicb.2018.00793 (PMC5945888; doi:10.3389/fmicb.2018.00793)
Supplement: Supplementary file 1 [file Presentation_1.pptx]

## Slide 1
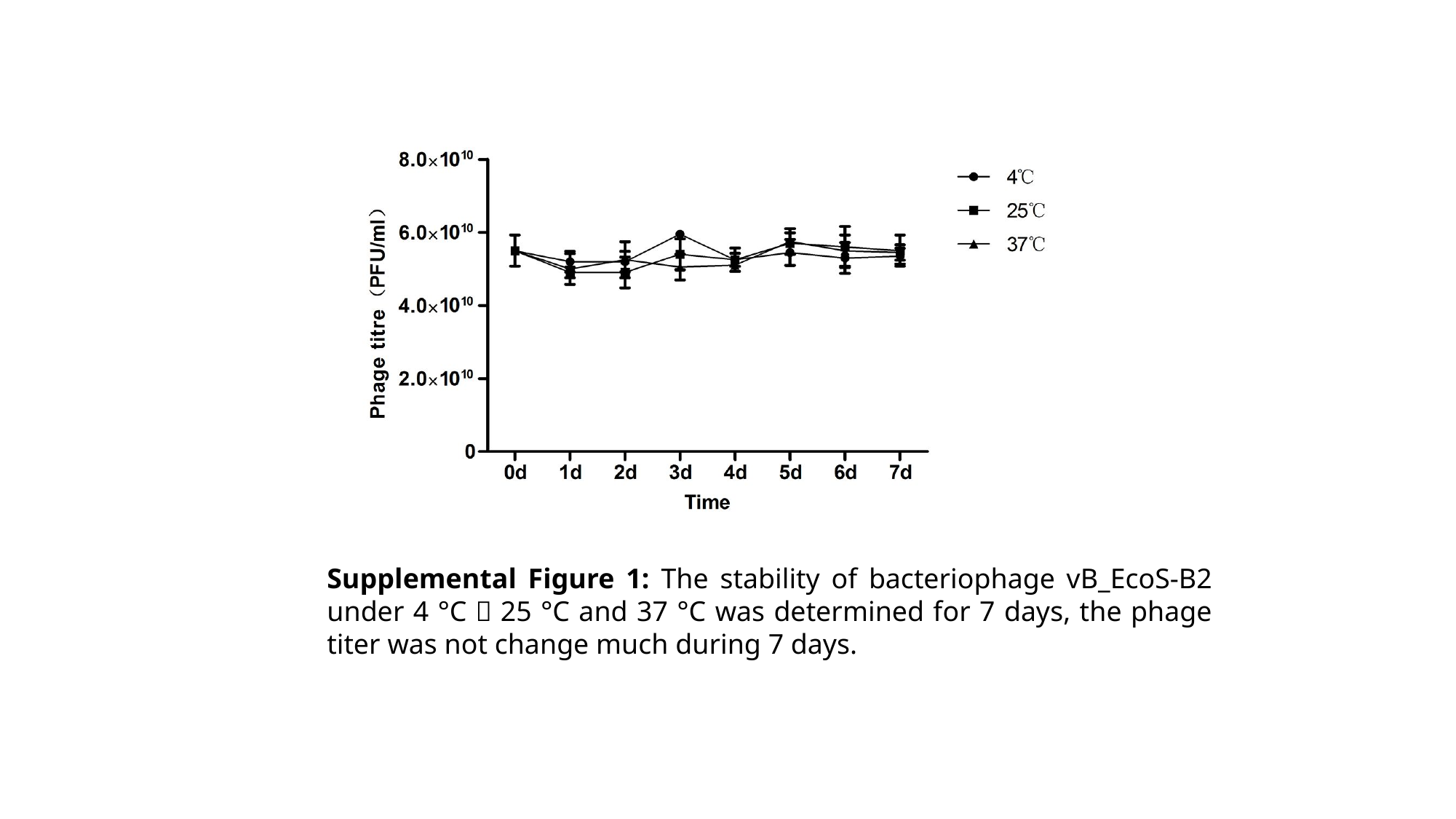

Supplemental Figure 1: The stability of bacteriophage vB_EcoS-B2 under 4 °C，25 °C and 37 °C was determined for 7 days, the phage titer was not change much during 7 days.

## Slide 2
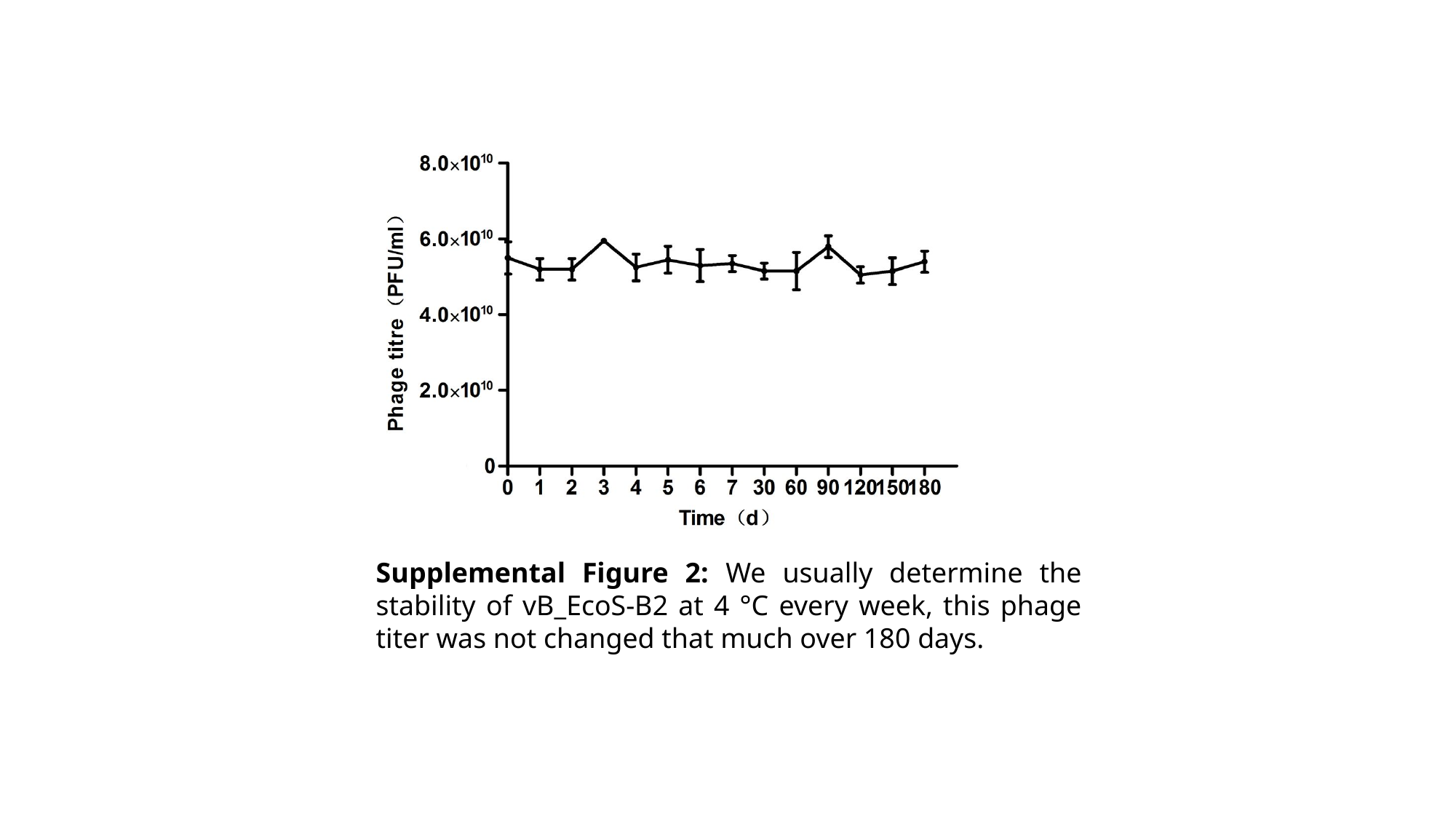

Supplemental Figure 2: We usually determine the stability of vB_EcoS-B2 at 4 °C every week, this phage titer was not changed that much over 180 days.

## Slide 3
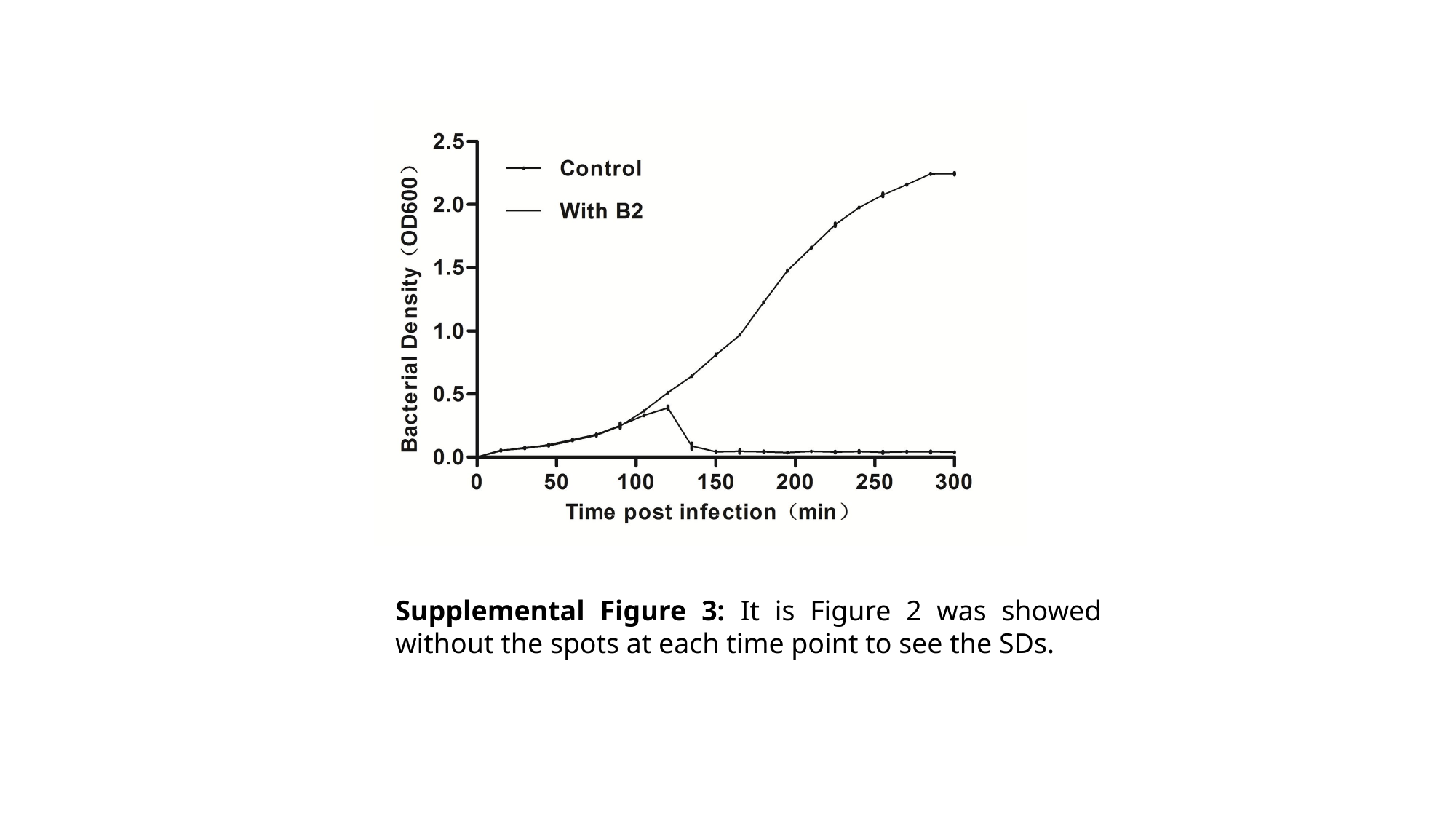

Supplemental Figure 3: It is Figure 2 was showed without the spots at each time point to see the SDs.
